# The Value of Real-Time Shear Wave Elastography before and after Rehabilitation of Upper Limb Spasm in Stroke Patients

**DOI:** 10.1155/2020/6472456

**Published:** 2020-08-18

**Authors:** Jun Liu, Huijuan Pan, Yong Bao, Yanna Zhao, Li Huang, Weiwei Zhan

**Affiliations:** ^1^Department of Ultrasonic Diagnosis, Rui Jin Hospital, Shanghai Jiao Tong University School of Medicine, China; ^2^Department of Rehabilitation Medicine, Shanghai Ruijin Rehabilitation Hospital, China; ^3^Department of Ultrasonic Diagnosis, Shanghai Ruijin Rehabilitation Hospital, China

## Abstract

**Objective:**

Our goal was to evaluate the efficacy of muscle spasm before and after rehabilitation by comparing shear wave propagation velocity (SWV) and Young's modulus (YM) in the normal and spastic biceps brachii in stroke patients.

**Methods:**

A study of 60 stroke patients with upper limb spasm was performed; these patients were admitted from April 2018 to September 2019. The modified Ashworth scale (MAS) scores of the spastic biceps brachii before and after rehabilitation treatment were compared. SWV and YM on the spastic and normal biceps brachii before rehabilitation treatment, SWV and YM on the spastic and normal biceps brachii after rehabilitation treatment, and SWV and YM on the spastic biceps brachii before and after rehabilitation treatment were compared. Whether SWV and YM on the spastic biceps brachii are related to MAS was compared.

**Results:**

There was a statistically significant difference in SWV and YM between the normal and spastic biceps brachii before (*P* < 0.01) and after (*P* < 0.05) the rehabilitation treatment. There was no statistically significant difference in SWV and YM in the normal biceps brachii before and after the rehabilitation treatment (*P* > 0.05). There was a statistically significant difference in SWV and YM in the spastic biceps brachii before and after the rehabilitation treatment (*P* < 0.01). SWV and FM of the spastic biceps brachii are correlated with MAS before and after rehabilitation treatment, and the correlation coefficient for SWV was 0.563 and 0.605 for YM (*P* < 0.05).

**Conclusion:**

SWE can be used as a means of assessment before and after rehabilitation treatment.

## 1. Introduction

Stroke is characterized by high incidence, and the patients have high disability rates. A large number of studies have shown that the incidence of upper limb spasm is significantly higher than that of the lower limb, with a more difficult rehabilitation [1–2]. Spasticity is a dyskinesia characterized by speed-dependent hypertonic stretch reflex and tendon hyperreflexia caused by increased excitatory reflex after upper motor neuron injury [3]. Spasticity is one of the main complications of stroke, which can cause muscle contraction, abnormal posture, pain, and joint contracture in patients, resulting in abnormal movement patterns that affect patients' daily life functions and restrict hemiplegic limbs.

At present, the assessment of dysfunction in patients with upper limb spastic paralysis is mostly based on subjective scale assessment, which is inaccurate and not sufficiently sensitive [4]. Muscle cramps generally manifest as increased muscle stiffness, which is positively correlated with active or passive muscle strength. Therefore, detecting changes in muscle stiffness can help assess changes in muscle strength. Real-time shear wave ultrasound (SWE) elastography can be used to evaluate muscle stiffness [5–9]. Assessing muscle stiffness is important for the selection and adjustment of rehabilitation treatment plans and assessment of rehabilitation effects and prognosis [10]. Therefore, the present study is aimed at evaluating the efficacy of muscle spasm before and after rehabilitation by comparing shear wave propagation velocity (SWV) and Young's modulus (YM) in the normal and spastic biceps brachii in stroke patients.

## 2. Materials and Methods

### 2.1. Patients

All study procedures involving human participants were in accordance with the ethical standards of the institutional and national research committee and the 1964 Declaration of Helsinki and its later amendments or comparable ethical standards. This study was approved by the hospital institutional review board. Oral informed consent was obtained from all individual participants included in the study, which was approved by the hospital institutional review board. Sixty stroke patients with hemiplegia and one-sided upper limb spasms were selected between April 2018 and September 2019. Participants included 36 males and 24 females. Patient ages ranged from 56 to 78 years, with an average of 66 ± 7.46 years. The course of stroke ranged from 1 to 6 months, with an average of 6.6 months. There were 48 cases of cerebral infarction, 12 cases of brain bleeding, 35 cases of left hemiplegia, and 25 cases of right hemiplegia. Patients with no upper limb spasm served as the control group.

### 2.2. Inclusion Criteria

The inclusion criteria were as follows: (1) patients with unilateral hemiplegia after the first stroke between 1 and 6 months, (2) cerebral hemorrhage or cerebral infarction confirmed by computer tomography or magnetic resonance imaging, (3) upper limb spasm on one side and no previous myogenic disease, (4) patients with elevated upper muscle tone and upper limbs who were able to remain in a stretched position and the modified Ashworth scale (MAS) graded 0–3, and (5) patients who can cooperate with the examination and assessment.

### 2.3. Exclusion Criteria

The exclusion criteria were as follows: (1) dystonia, such as Parkinson's syndrome; (2) paralysis of bilateral limbs, such as increased muscle tone due to spinal cord injury; (3) complications of serious medical illness or mental or cognitive impairment, such that the patient cannot cooperate with examination; and (4) MAS graded 4 and patient with stiff muscles who was unable to stretch and flex.

### 2.4. MAS Assessment

MAS scores were as follows: level 0—0 points, level 1—1 point, level 1+—2 points, level 2—3 points, level 3—4 points, and level 4—5 points. The total MAS scores in the spastic side before and after the rehabilitation treatment were compared.

### 2.5. Equipment and Methods

This study was performed using a sonography scanner incorporating a 9L probe (LOGIQ E9) with a frequency of 9 MHz.

All patients were placed in a supine position, with the upper limbs of the spastic side positioned at 90 degrees relative to the body and biceps brachii in a stretched position. First, the probe was used to find the largest cross-section of the musculoabdomen biceps, and then it was rotated 90 degrees, parallel to the direction of the muscle bundle. SWE was then performed within an ~10 × 10 mm area of interest and a depth of 1–3 cm. After the elastic image was stabilized, the Q-BOX function was frozen to measure the SWV (m/s) and YM (kPa) of the muscle tissue in the region of interest. The measuring circle diameter was 2 mm, and the same location was measured three times to obtain an arithmetical average value. After the spastic side test was completed, the normal side test was performed according to the above method. The above examination after the rehabilitation treatment was repeated, and the bilateral SWV and YM were compared. The SWV and YM correlation was analyzed using MAS.

### 2.6. Rehabilitation Treatment

Routine rehabilitation treatment utilized Bobath technology and relearning exercises once a day using one-on-one training with a professional rehabilitation therapist 45 min each time. The remaining training was practiced by the patients themselves. Functional electrical stimulation used two electrode plates placed on the triceps brachii to trigger elbow extension (one electrode plate was placed on the radial head and the other on the dorsal muscles of the forearm to initiate back extension of the hand). One electrode plate was placed on the back of the deltoid muscle and the other was placed on the supraspinatus muscle to trigger shoulder lift and abduction based on patient tolerance. The treatment was performed for 30 min each time, two times a day for a total treatment time of six weeks.

### 2.7. Statistical Methods

The data were expressed as the mean value ± one standard deviation using SPSS19.0 software. An independent sample nonparametric test was used to compare between and within groups. Spearman's test was used for the correlation analysis of SWV and YM with MAS. *P* < 0.05 was considered statistically significant.

## 3. Results

### 3.1. MAS Assessment

A total of 12 people were in MAS level 1, 17 in MAS level 1+, 22 in MAS level 2, and nine in MAS level 3 before the rehabilitation treatment. There were six people in MAS level 0, 16 in MAS level 1, 17 in MAS level 1+, 17 in MAS level 2, and four in MAS level 3 after the rehabilitation treatment. Statistically significant differences were present in the MAS median before and after the rehabilitation treatment (*P* < 0.05) (Table 1).

### 3.2. SWE Assessment

**Figure 1:**
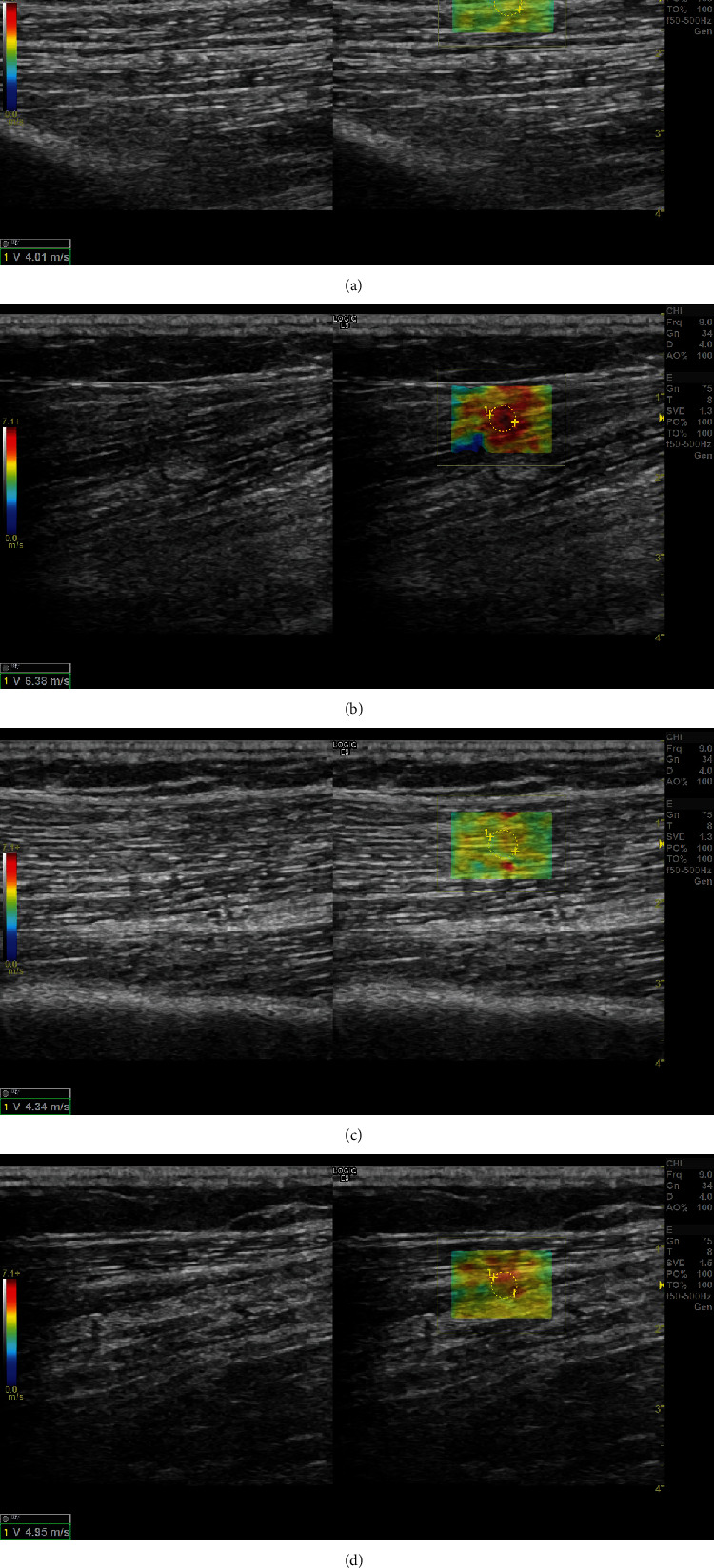
Male, 69 years old, stroke patient with right hemiplegia. SWV was 4.01 m/s (a) on the normal side (left side), and SWV was 6.36 m/s (b) on the spastic side (right side) before rehabilitation treatment. After six weeks of rehabilitation treatment, SWV was 4.34 m/s (c) on the normal side (left side) and 4.95 m/s (d) on the spastic side (right side).

SWV in the normal biceps brachii was 4.12 ± 1.25 m/s (Figure 1(a)) and 6.58 ± 1.16 m/s in the spastic biceps brachii (Figure 1(b)) before the rehabilitation treatment. YM in the normal biceps brachii was 47.16 ± 7.56 kPa and 123.34 ± 25.74 kPa in the spastic biceps brachii before the rehabilitation treatment. SWV in the normal biceps brachii was 4.34 ± 1.32 m/s (Figure 1(c)) and 4.95 ± 1.02 m/s in the spastic biceps brachii (Figure 1(d)) after the rehabilitation treatment. YM in the normal biceps brachii was 50.58 ± 10.54 kPa and 72.08 ± 9.86 kPa in the spastic biceps brachii after the rehabilitation treatment. There was a statistically significant difference in SWV and YM between the normal and spastic biceps brachii before (*P* < 0.01) and after (*P* < 0.05) (Table 2) the rehabilitation treatment. There was no statistically significant difference in SWV and YM in the normal biceps brachii before and after the rehabilitation treatment (*P* > 0.05). There was a statistically significant difference in SWV and YM in spastic biceps brachii before and after the rehabilitation treatment (*P* < 0.01) (Table 3).

### 3.3. Correlation Analysis between SWE and MAS

Correlation analysis between SWV and YM in the spastic biceps brachii was performed using MAS before and after the rehabilitation treatment. A statistically significant difference was present (*P* < 0.05). The correlation coefficient for SWV was 0.563 and 0.605 for YM (Table 4).

## 4. Discussion

After a stroke, limb spasms are often due to central nervous injury or a lesion in the conduction system, which leads to excessive release of lower motor neurons and consequent imbalances in the active and antagonist limb muscles. In the early stages of limb movements, joint movements are slow and coarse movements are difficult to control accurately. If a spasm cannot be effectively treated for a prolonged period of time, it will continue to increase, resulting in limb muscle atrophy, tendon contracture, and joint deformity. In severe cases, heterotopic ossification of the muscle tissue around the contracted joints will occur, aggravating limb dysfunction and causing significant difficulties during later rehabilitation treatment. Therefore, when the patient's limbs begin to show a tendency to spasm, an accurate assessment should be made as early as possible and effective treatment measures should be taken. These steps are of great significance to the overall patient rehabilitation [11–12].

The diagnostic assessment of spasticity is based on the experience of a clinically evaluated physician or physical therapist. The MAS is the most commonly used assessment scale. It is the most widely used method in clinical practice, because this scale is easy to evaluate and does not require additional equipment and technical processing. The present study used MAS to evaluate the curative effect before and after rehabilitation of the upper limb spasm and to reflect the effect of rehabilitation treatment [13]. There were statistically significant differences in the total MAS score before and after the rehabilitation treatment (*P* < 0.05). However, MAS is often affected by factors, such as an examiner's experience, subjective individual differences, and understanding of the scale standards. It cannot accurately quantify changes in muscle tension, because an increase in muscle tension involves not only resistance felt by the limbs but also physical tissue properties and elasticity inside the muscle or connective tissue. Clinical evaluations of muscle tension are related only to the classification of resistance felt by the flexion and extension of the limbs. To date, there are no relevant data pertaining to specific changes in the elasticity inside the muscle or connective tissue and their relationship with classification of increased muscle tone [4].

Acoustic pulses emitted by the ultrasound probes are focused at different depths in the tissue to generate transverse shear waves. According to the Mach cone principle [14–15], a quantitative analysis system can be used to calculate the physical quantity of YM that reflects SWV in the tissue. The relationship between YM and SWV is as follows: *E* = 3*pc*^2^ (*E*: YM; *c*: SWV; *p*: tissue density). The harder the structure is, the greater its density is, and the faster the SWV is, the higher its YM is [16]. In the present study, SWV in the normal biceps brachii was 4.12 ± 1.25 m/s and 6.58 ± 1.16 m/s in the spastic biceps brachii before the rehabilitation treatment. YM in the normal biceps brachii was 47.16 ± 7.56 kPa and 123.34 ± 25.74 kPa in the spastic biceps brachii before the rehabilitation treatment. There was a statistically significant difference in SWV and YM between the normal and spastic biceps brachii (*P* < 0.01).

The structural characteristics of muscles determine their biomechanical properties and are important determinants of muscle function [17]. In the present study, SWV and YM in the normal biceps brachii before the rehabilitation treatment were 4.12 ± 1.25 m/s and 47.16 ± 7.56 kPa, respectively. After the rehabilitation treatment, SWV and YM in the normal biceps brachii were 4.34 ± 1.32 m/s and 50.58 ± 10.54 kPa, respectively. Therefore, the muscle fiber structure of normal muscles was intact and there were no statistically significant differences in SWV and YM in normal biceps brachii before and after the rehabilitation treatment (*P* > 0.05). After a stroke, a decrease in muscle fibers, cross-sectional muscle area, and proliferation of connective tissue around the muscle will lead to changes in muscle stiffness [18]. By comparing biopsies of spastic and normal muscles in patients with cerebral palsy, Fridén and Lieber [19] found that spastic muscle cells in patients with cerebral palsy are shorter in resting sarcomeres than in normal myocytes, have a higher elastic modulus, and have the most noticeable effect within the actin molecule. It can be inferred that shortening of the sarcomere is one of the reasons that leads to a greater stiffness in the hemiplegic side of the muscle after stroke. In the present study, SWV and YM in the spastic biceps brachii increased after a stroke and were statistically different compared to the normal side. This change in stiffness with muscle stretching is similar to that described by Koo et al. [20]. According to the myofilament theory, some actin sites bind to the transverse bridge, which is directly related to the myofilament. The SWV and YM changes are in agreement with molecular biological characteristics of the skeletal muscle and theoretically can reflect skeletal muscle biomechanical characteristics. In addition, the change in biceps stiffness with muscle stretching is greater on the stroke side, which may be related to a gradual decrease in muscle fibers and a decrease in the muscle cross-sectional area in the hemiplegic limb after stroke.

In the present study, both SWV and YM in the biceps brachii were decreased after the rehabilitation treatment compared to those before rehabilitation. SWV in the spastic biceps brachii was 6.58 ± 1.16 m/s, and YM was 123.34 ± 25.74 kPa before the rehabilitation treatment. After rehabilitation, SWV in the spastic biceps brachii was 4.95 ± 1.02 m/s and YM was 72.08 ± 9.86 kPa. There was a statistically significant difference in SWV and YM in the spastic biceps brachii before and after the rehabilitation treatment (*P* < 0.01). However, SWV and YM were still higher compared to the normal side after the rehabilitation treatment. SWV in the normal biceps brachii was 4.34 ± 1.32 m/s and 4.95 ± 1.02 m/s in the spastic biceps brachii after the rehabilitation treatment. YM in the normal biceps brachii was 50.58 ± 10.54 kPa and 72.08 ± 9.86 kPa in the spastic biceps brachii after the rehabilitation treatment. There was a statistically significant difference in SWV and YM in the spastic biceps brachii after the rehabilitation treatment (*P* < 0.05), which may be due to the short duration of the study. It is possible that SWV and YM in the biceps brachii on the spastic side may be similar to those on the normal side. Nevertheless, a decrease in SWV and YM indicates that the degree of muscle spasm was improved and that rehabilitation treatment was effective. In the present study, SWV and YM in the spastic biceps brachii were correlated with MAS before and after the rehabilitation treatment, with SWV and YM correlation coefficients of 0.563 and 0.605, respectively (*P* < 0.05). These data provide more objective evidence for rehabilitation physicians to determine whether the patient rehabilitation treatment is effective.

In conclusion, SWE can be used as a quantitative index to analyze changes in the muscle structure and function after stroke and as a means of assessment before and after the rehabilitation treatment.

## Figures and Tables

**Table 1:** Comparison of MAS median before and after rehabilitation treatment.

	Before	After	*P* value
MAS median	68.06	52.94	*P* < 0.05

MAS: modified Ashworth scale.

**Table 2:** Comparison of SWV and YM between normal and spastic biceps brachii before and after rehabilitation treatment.

Rehabilitation treatment	Before			After		
	Spastic side	Normal side	*P* value	Spastic side	Normal side	*P* value
SWV	6.58 ± 1.16	4.12 ± 1.25	*P* < 0.01	4.95 ± 1.02	4.34 ± 1.32	*P* < 0.05
YM	123.34 ± 25.74	47.16 ± 7.56	*P* < 0.01	72.08 ± 9.86	50.58 ± 10.54	*P* < 0.05

SWV: shear wave propagation velocity. YM: Young's modulus.

**Table 3:** SWV and YM comparison in normal and spastic biceps brachii before and after rehabilitation treatment.

Rehabilitation treatment	Normal biceps brachii			Spastic biceps brachii		
	Before	After	*P* value	Before	After	*P* value
SWV	4.12 ± 1.25	4.34 ± 1.32	*P* > 0.05	6.58 ± 1.16	4.95 ± 1.02	*P* < 0.01
YM	47.16 ± 7.56	50.58 ± 10.54	*P* > 0.05	123.34 ± 25.74	72.08 ± 9.86	*P* < 0.01

SWV: shear wave propagation velocity. YM: Young's modulus.

**Table 4:** SWV and YM correlation analysis using MAS on the spastic side.

	MAS	
	*P* value	Correlation coefficient
SWV	*P* < 0.05	0.563
YM	*P* < 0.05	0.605

SWV: shear wave propagation velocity. YM: Young's modulus. MAS: modified Ashworth scale.

## Data Availability

The data used to support the findings of this study are included within the article.
